# Gait oscillation analysis during gait and stair-stepping in elder patients with knee osteoarthritis

**DOI:** 10.1186/s13018-019-1064-6

**Published:** 2019-01-16

**Authors:** Takatomo Mine, Masaya Kajino, Jun Sato, Seiichi Itou, Koichiro Ihara, Hiroyuki Kawamura, Ryutaro Kuriyama, Yasuhiro Tominaga

**Affiliations:** 1 Department of Orthopaedic Surgery, National Hospital Organization Kanmon Medical Center, 1-1 Choufusotoura Shimonoseki, Yamaguchi, 752-8510 Japan; 2 Department of Rehabilitation, National Hospital Organization Kanmon Medical Center, Yamaguchi, Japan

**Keywords:** Gait oscillation, Gait, Stair-stepping, Osteoarthritis of the knee

## Abstract

**Background:**

Evaluation of knee and lower limb function alone is not sufficient to assess gait. For accurate assessment of gait abnormality, gait oscillation should also be measured. The goal of this analysis was to assess the influence of the knee joint on gait oscillation during gait and stair-stepping in patients with osteoarthritis of the knee.

**Methods:**

In 33 patients diagnosed with knee osteoarthritis and 33 healthy adults as the control group, we examined acceleration (anterior and lateral directions) and gait barycentric factors (single-support phase and ratio of center of gravity maximum values) during gait and stair-stepping.

**Results:**

Acceleration in the anterior direction in the sacral region was greater in healthy adults than in osteoarthritis (OA) patients during gait and stair-down. Acceleration in the anterior direction in the dorsal vertebral region was greater in OA patients than in healthy adults during (up and down) stair-stepping. Acceleration in the lateral direction in the sacral region was greater in healthy adults than in OA patients during stair-up. Acceleration in the lateral direction in the dorsal vertebral region was greater in OA patients than in healthy adults during stair-stepping. The single-support phase was close to 1 for gait and stair-stepping in healthy adults and OA patients. The single-support time was largely the same for gait and stair-stepping in healthy adults. On the other hand, the single-support time was longer for stair-stepping than for gait in OA patients. The ratio of the center of gravity maximum values was greater for the sacral region than for the dorsal vertebral region. There was a significant difference in the stair-stepping ratio of the center of gravity maximum values between healthy adults and OA patients for the sacral region.

**Conclusion:**

We considered that knee OA influenced acceleration in the anterior and lateral direction in the dorsal vertebral and the ratio of the center of gravity maximum values on gait oscillation.

## Introduction

Gait is one of the main activities of daily living. Knee function is very important in gait. Knee function is decreased by pain and knee deformity in elder patients with knee osteoarthritis (OA). It is important to assess the effect on gait by knee functional decline in elder patients with knee OA. The methods for evaluating knee function are clinical and radiological assessment. However, evaluation of knee function alone is not sufficient for gait assessment. Therefore, there are many reports concerning gait analysis [1–4]. Gait analysis provides a non-invasive and convenient method for studying full-body kinematics and kinetics over large fields of measurement. Many gait analyses have been reported concerning patients with knee OA [5–10]. There are a lot of parameters such as velocity, stride length, step width, swing/stance ratio, single-limb support time, and maximum angular velocity to assess the gait. However, there are no standardized objective parameters that can evaluate body balance during walking. Recently, gait analysis using an accelerometer has been performed step by step [11, 12]. The significance of the acceleration measurement during gait is to assess the smoothness of gait activity objectively. We considered that from a change in acceleration during gait, we can evaluate gait oscillation objectively. Recently, we assessed gait oscillation during gait and stair-stepping in elder patients after total knee arthroplasty (TKA) with a 2-point gait oscillometer MVP-WS2-S. This device can display visualization of body movement while walking [13]. However, there is no a “normal” value for gait oscillation. Therefore, we considered a comparison with healthy adults who do not have any lower extremity injuries to be necessary. The goal of this study was to assess gait oscillation during gait and stair-stepping in elder patients who have osteoarthritis of the knee through comparison with healthy adults who do not have any lower extremity injuries.

## Materials and methods

Thirty-three patients diagnosed with knee osteoarthritis (OA) and 33 healthy adults (as the control group) participated in this study. The OA group included 20 female and 13 male patients. Their average age was 75.1 (range 63–87) years. Crystal-induced inflammation or elderly onset rheumatoid arthritis in the joints was excluded. These participants had no histories of lower limb joint surgery, neurologic impairments, or declared any incidence of arthritis that had undergone conservative treatment. The healthy adult group included 13 females and 20 males, and their average age was 32.8 (range 20–49) years. Healthy adult group consisted of medical staff at our hospital at the time of the measurements. These participants had no histories of lower limb joint surgery, neurologic or musculoskeletal impairments, and no declared incidence of arthritis.

Clinical evaluations were made according to the knee-rating scale of the Hospital for Special Surgery (HSS). Radiological evaluations were performed using the Kellgren-Lawrence classification (K-L grade) (Table 1).

**Table 1:** Patient characteristics

	OA patient	Healthy adult
Mean age	75.6 ± 9.8	32.8 ± 10.6
Gender (male/female)	13/20	20/13
Mean body mass index	24.2 ± 7.0	21.7 ± 3.1
HSS score	68.4 ± 7.2	100
K-L grade	Grade 2:9, 3:24	–

Gait oscillation analyses in this study were performed by examining acceleration (anterior and lateral directions) and gait barycentric factors (single-support phase and ratio of the center of gravity maximum values) in gait and stair-stepping. These results between the OA patient group and healthy adult group were compared using one-factor repeated measures analysis of variance. Statistical analysis was performed using R2.8.1. The Shaffer method was used for the multiple comparison procedure [14].

**Fig. 1:**
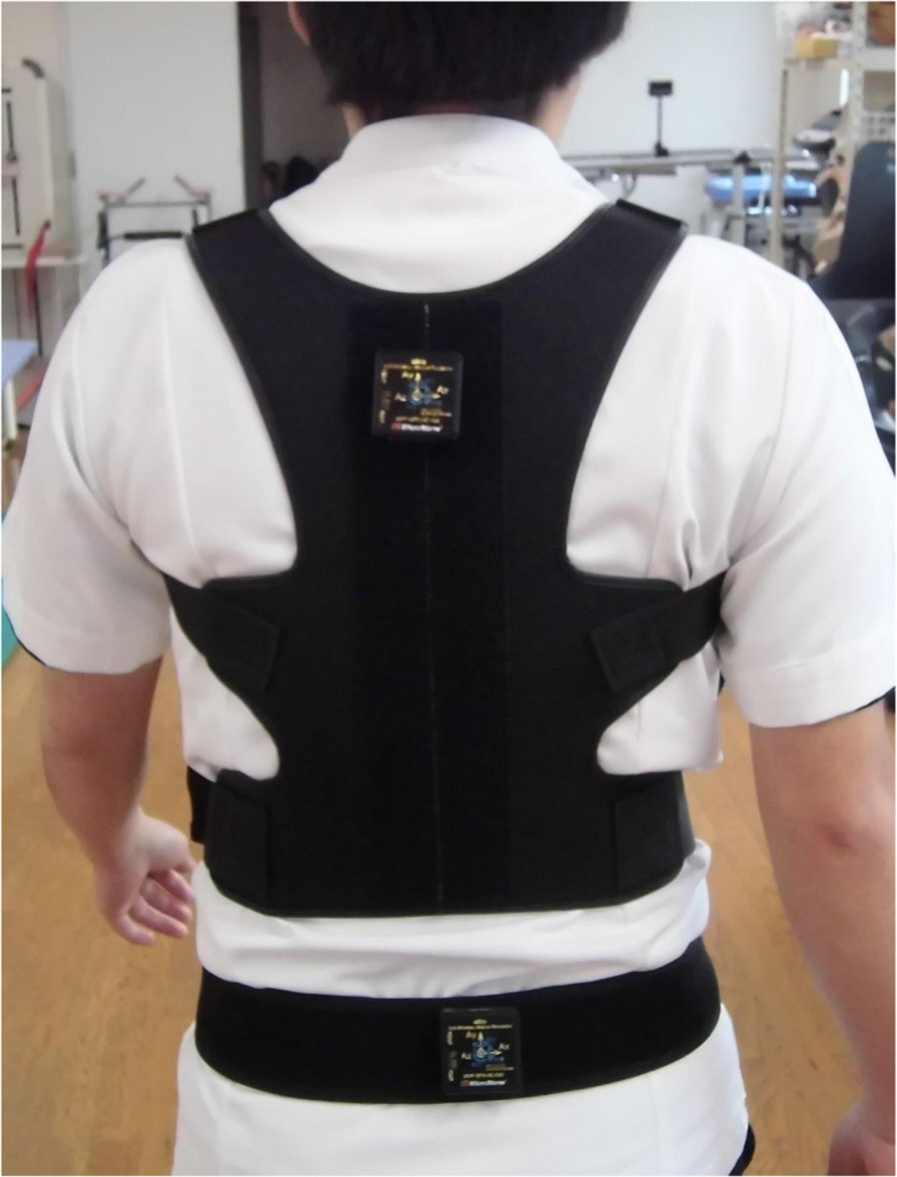
Two-point gait oscillometer MVP-WS2-S. Two compact wireless sensors were attached to the dorsal vertebral region and the sacral region with the specified harness and belt

**Fig. 2:**
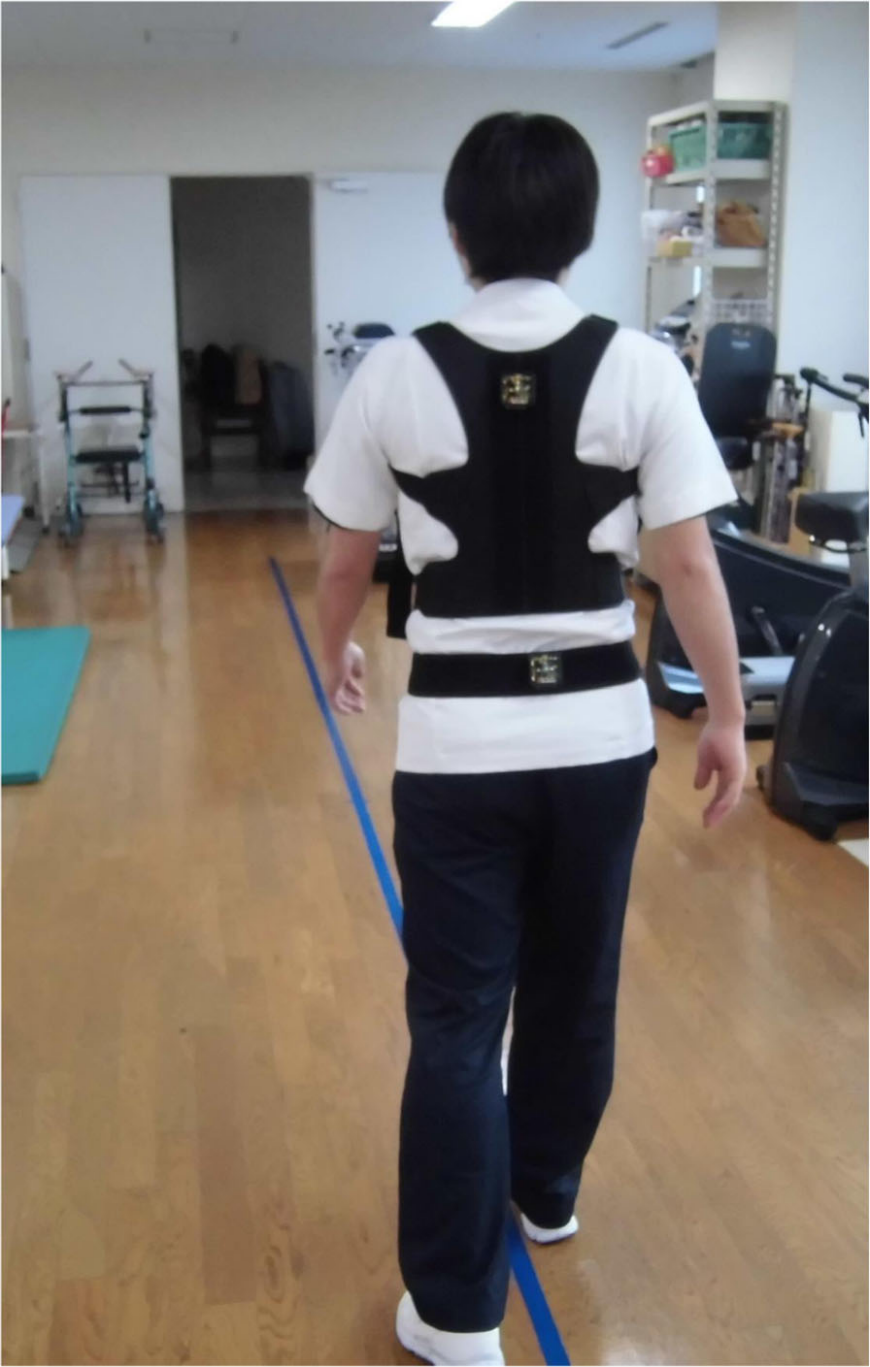
Assessment of gait oscillation during gait (10 m)

Using a 2-point gait oscillometer MVP-WS2-S (Microstone Corp., Japan), we assessed gait oscillation during gait (10 m) and stair-stepping. Two compact wireless sensors were attached to the dorsal vertebral region and the sacral region with a harness and belt (Figs. 1 and 2). Three successful measurements were recorded, and the recordings were used for the analysis.

For the ratio of the center of gravity maximum values, the barycentric transfer during gait was plotted in a figure eight on a graph. In this graph, the ratio of the center of gravity maximum values was defined as the maximum of the vertical axis/the maximum of the horizontal axis and the index of the barycentric transfer during gait.

## Results

The mean and standard deviation values of the observed variables are shown in Table 2. There was a significant difference between the OA group and the healthy adult group in acceleration in the anterior direction during gait and stair-down in the sacral region (*p* < 0.01) and during stair-up and stair-down in the dorsal vertebral region (*p* < 0.01).

**Table 2:** Parameters between healthy adults and OA patients during gait and stair-stepping

	Gait		Step-up		Step-down		
	Healthy adults	OA patients	Healthy adults	OA patients	Healthy adults	OA patients	
Acceleration (m/s^2^)							
To the anterior							
Sacral region	8.19 ± 1.91	6.91 ± 3.08	7.14 ± 2.42	7.26 ± 2.96	7.62 ± 1.88	6.17 ± 2.52	Gait; healthy > OA**, down; healthy>OA**
Dorsal vertebral region	4.97 ± 1.56	5.60 ± 1.61	4.65 ± 1.21	6.98 ± 1.77	3.81 ± 1.40	6.75 ± 2.89	Stair-stepping; healthy < OA**
To the OA direction							
Sacral region	5.88 ± 1.92	6.04 ± 4.35	4.35 ± 1.72	3.35 ± 1.87	5.54 ± 2.61	5.64 ± 3.78	Up; healthy > OA**
Dorsal vertebral region	3.71 ± 1.02	4.09 ± 2.45	3.14 ± 1.02	2.96 ± 1.93	3.92 ± 1.82	4.37 ± 2.60	
To the contralateral direction							
Sacral region	5.86 ± 2.21	5.04 ± 2.57	4.33 ± 1.72	3.47 ± 1.83	5.24 ± 2.73	5.42 ± 3.06	Up; healthy > OA*
Dorsal vertebral region	3.20 ± 1.33	3.72 ± 1.39	2.45 ± 0.89	2.87 ± 1.72	2.88 ± 1.38	3.65 ± 2.09	Stair-stepping; healthy <OA*
Single-support time (s)							
OA side	0.52 ± 0.05	0.70 ± 0.23	0.57 ± 0.06	1.38 ± 1.20	0.55 ± 0.05	1.17 ± 0.57	Gait, stair-stepping; healthy < OA**
Contralateral side	0.53 ± 0.05	0.70 ± 0.20	0.58 ± 0.06	1.10 ± 0.55	0.55 ± 0.07	1.23 ± 0.80	Gait, stair-stepping; healthy < OA**
Single-support phase	0.99 ± 0.09	1.00 ± 0.17	0.99 ± 0.06	1.02 ± 0.29	0.99 ± 0.10	1.03 ± 0.38	
Ratio of the center of gravity maximum values							
Sacral region	0.76 ± 0.30	0.95 ± 0.47	0.75 ± 0.32	1.03 ± 0.69	0.80 ± 0.32	0.91 ± 0.73	Down; healthy < OA**
Dorsal vertebral region	0.64 ± 0.24	0.70 ± 0.50	0.40 ± 0.15	0.92 ± 0.75	0.39 ± 0.17	0.89 ± 0.68	Stair-stepping; healthy < OA**

*p < 0.05, **p < 0.01

There was a significant difference between the OA group and the healthy adult group in acceleration in the lateral direction (OA side) during stair-up in the sacral region (*p* < 0.01). There was no significant difference between the OA group and the healthy adult group in acceleration in the lateral direction during gait and stair-up and stair-down in the dorsal vertebral.

There was a significant difference between the OA group and the healthy adult group in acceleration in the contralateral direction during stair-up in the sacral region (*p* < 0.05) and during stair-up and stair-down in the dorsal vertebral regions (*p* < 0.05).

There was a significant difference between the OA group and the healthy adult group in the single-support phase during gait and stair-stepping values (*p* < 0.01).

There was a significant difference between the OA group and the healthy adult group in ratio of the center of gravity maximum values during stair-down values in the dorsal vertebral region (*p* < 0.01) and during stair-stepping values in the sacral region (*p* < 0.01).

## Discussion

In OA patients, gait is disturbed by knee pain and deformity. However, improvement of knee function alone is not sufficient to improve gait. It is very important to assess gait oscillation, including the function of the trunk. In this study, we assessed the difference in gait oscillation during gait and stair-stepping in OA patients and healthy adults as a control group. Acceleration in the anterior direction in the sacral region during gait and stair-down and in the lateral region in the sacral region during stair-down was greater in healthy adults than in OA patients. The ratio of the center of gravity maximum values was greater for the sacral region than for the dorsal vertebral region. This is more obvious in healthy adults than in OA patients. From these findings, we considered that gait and stair-stepping might be performed mainly on the pelvic girdle more in healthy adults than in OA patients. On the other hand, acceleration in the anterior direction in the dorsal vertebral region during stair-stepping was greater in OA patients than in healthy adults. The ratio of the center of gravity maximum values during stair-stepping was greater in OA patients than in healthy adults. Turcot reported that the knee OA patients increase trunk flexion angle and decrease knee flexion moment during stair-stepping, and they compensate for stair-stepping motion with anteroposterior movement of the upper trunk [15]. Therefore, we considered that gait oscillation in the anterior direction might increase more in OA patients than in healthy adults during stair-stepping. Acceleration in the lateral direction in the dorsal vertebral region during stair-stepping was greater in OA patients than in healthy adults. The ratio of the center of gravity maximum values was smaller in healthy adults than in OA patients. From these findings, we considered that gait oscillation in the lateral direction during stair-stepping might increase more in OA patients than in healthy adults. To perform suitable transfer of center of gravity to the supporting lower limb, the reinforcement of a hip joint abductor and the trunk function by exercise therapy are very important. The single-support phase was close to 1 during gait and stair-stepping in healthy adults and OA patients. The single-support time was largely the same during gait and stair-stepping in healthy adults. On the other hand, the single-support time was longer during stair-stepping than during gait in OA patients. From these findings, we considered that gait and stair-stepping exercise were slower in OA patients and that this might relieve their knee pain. Therefore, the single-support phase may be close to 1 for gait and stair-stepping in OA patients. OA is thought to be caused by degenerative changes in articular cartilage and secondary proliferative changes in cartilage and bone, resulting in pain, joint deformity, and functional disorders with aging. Klitgaad reported that muscle mass, muscle strength, and muscle contractility decline with age [16]. Judge reported older adults take shorter steps, spend more time in single support, and walk with their pelvis rotated anteriorly, hip slightly flexed, and toes pointing out [17]. Therefore, in OA patients, not only knee function, but also influence by aging have to be considered. However, we reported the assessment of gait oscillation in elder OA patients that received unilateral TKA [13]. The values of gait oscillation were different between healthy adults and elder patients after TKA, but gait and stair-stepping were performed mainly on the pelvic girdle. Gait oscillation seemed to show the same tendency. We consider that, according to gait oscillation, the influence of aging may be small.

There were some limitations to our study. The small number of patients weakens the statistical power of the results. Further investigation with a larger sample size is needed to obtain more clinical data. The participants significantly differed in the percentage of each sex within each group. There was no significant difference in the data between male and female for either OA patients or for healthy adults. However, detection power was small. A further limitation was that there was a difference in subjects’ ages. Since the OA patients were older than the healthy adults, the influence of age may have to be considered. In addition, standard error of measurements may have decreased the generalizability of this study. Despite these limitations, the present study contributes to our current understanding of gait oscillation.

## Conclusion

Acceleration in the anterior direction in the sacral region during gait and stair-down and in the dorsal vertebral region during stair-stepping was greater in healthy adults than in OA patients. The ratio of the center of gravity maximum values was greater for the sacral region than for the dorsal vertebral region. This is more obvious in healthy adults than in OA patients. Acceleration in the lateral direction in the sacral region during stair-stepping was greater in OA patients than in healthy adults. The ratio of the center of gravity maximum values was smaller in healthy adults than in OA patient. We considered that knee OA influenced acceleration in the lateral direction and the ratio of the center of gravity maximum values on gait oscillation.

## Abbreviations

OA: Osteoarthritis
TKA: Total knee arthroplasty
